# Microscopic Examination of Cold Spray Cermet Sn+In_2_O_3_ Coatings for Sputtering Target Materials

**DOI:** 10.1155/2017/4058636

**Published:** 2017-01-09

**Authors:** M. Winnicki, A. Baszczuk, M. Rutkowska-Gorczyca, M. Jasiorski, A. Małachowska, W. Posadowski, Z. Znamirowski, A. Ambroziak

**Affiliations:** Wrocław University of Technology, Wyb. Wyspiańskiego 27, 50371 Wrocław, Poland

## Abstract

Low-pressure cold spraying is a newly developed technology with high application potential. The aim of this study was to investigate potential application of this technique for producing a new type of transparent conductive oxide films target. Cold spraying technique allows the manufacture of target directly on the backing plate; therefore the proposed sputtering target has a form of Sn+In_2_O_3_ coating sprayed onto copper substrate. The microstructure and properties of the feedstock powder prepared using three various methods as well as the deposited ones by low-pressure cold spraying coatings were evaluated, compared, and analysed. Produced cermet Sn+In_2_O_3_ targets were employed in first magnetron sputtering process to deposit preliminary, thin, transparent conducting oxide films onto the glass substrates. The resistivity of obtained preliminary films was measured and allows believing that fabrication of TCO (transparent conducting oxide) films using targets produced by cold spraying is possible in the future, after optimization of the deposition conditions.

## 1. Introduction

Transparent conducting oxide (TCO) is a heavily doped oxide semiconductor with high electrical conductivity and transparency over the visible spectral range. Due to their high conductivities and sufficiently large band gap (≥3 eV), the TCO films show also high reflectivity in the near infrared. Application in electronic and optoelectronic fields has found several TCO materials: tin oxide (SnO), zinc oxide (ZnO), indium oxide (In_2_O_3_), aluminium-doped zinc oxide (AZO), indium zinc oxide (IZO), and indium tin oxide (In_2_O_3_:Sn (ITO)). Among listed materials Sn-doped In_2_O_3_ thin films are desirable due to their lowest resistivity, highest transmission, and electrical conductivity properties that allow thinner, more flexible layers. Moreover, ITO films are stable and repeatable [1–9]. Therefore indium tin oxide (ITO) is one of the most widely used transparent conductive oxides (TCOs) for large-area coating applications in optoelectronics, such as liquid crystal displays, flat panel displays, plasma displays, touch panels, organic light-emitting diodes (OLEDs), electronic ink applications, photodiodes, solar cells, antistatic coatings, and other products requiring thin film coatings [1–13].

Several deposition processes have been developed to prepare ITO films, such as physical vapour deposition (PVD) (e.g., sputtering and vacuum evaporation), direct current (DC), and radio frequency (RF) sputtering, radio frequency (RF) ion plating, spray pyrolysis, sol-gel method, and chemical vapour deposition (CVD) [1–13]. Among these methods, the magnetron sputtering is widely used since the method is superior in its controllability and high deposition rate. Films deposited by this method show good uniformity over wide area on large sized substrates [14–18]. Magnetron sputtering requires an ITO sputtering target. The latter is a solid plate of high density ITO material. In a sputtering system, material to be deposited on a substrate is ejected from a target by bombardment of the target with ions.

During the deposition, the target material quality and performance directly affect the properties of the ITO films. Targets used in magnetron sputtering process are ceramic (In_2_O_3_-SnO_2_) or metallic (In-Sn alloy) [18]. Metallic indium tin alloy targets are sputtered with Ar-O_2_ mixture and require a special feedback control for keeping the film stoichiometry constant. Ceramic oxide targets are considered to provide higher performance compared to metallic targets. They are more uniform promoting the films with lower resistivity [6, 18]. However, ceramic oxide targets are prepared by hot pressing, where it is difficult to obtain uniform pressure across the target surface, leading to the problems of target warp or cracking [19–24]. What is more, such a target suffers from nonuniformity of density as well as nonuniformity of chemical and physical properties across the target body (caused by nonuniform distribution of tin ions and oxygen vacancies). It results in nonhomogenous sputtering across the target surface during the sputtering process. Moreover the formation of a black deposit, called a nodule and consisting mainly of In_2_O [25], on the target surface with an increase of the sputtering time is possible. The black deposit reduces the target utilization rate to much less than 30%, since mentioned surface irregularities adversely affect ITO thin film quality [21, 23]. These nodules usually possess a shape of a hillock, cone, or pyramid and tend to grow as the deposition run proceeds. Formation of nodules affects sputtering process by changing sputtering rate, angular distribution of sputtered atoms, enhanced arcing, and process drift and destabilization, which in turn result in defects and lead to poor quality sputtered films [1, 2, 10, 19–24, 26]. As a result the deposition process has to be interrupted cyclically in order to clean the target surface from nodules and debris. This causes an undesired downtime reducing production rate and so unquestionably formation of nodules on the target surface is highly undesirable.

**Figure 1:**
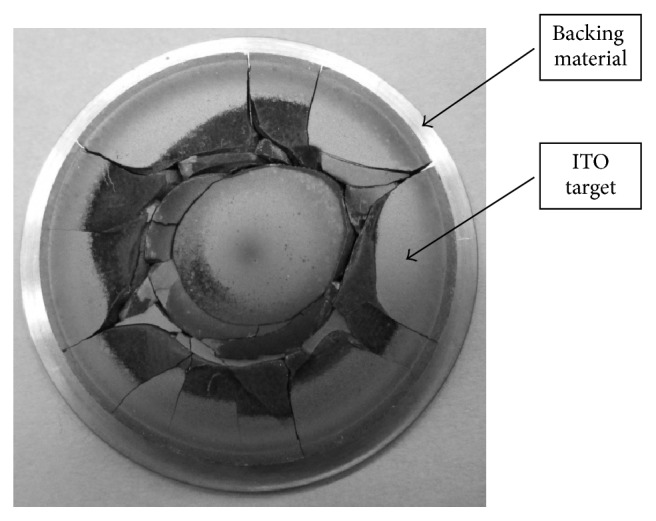
Commercially available target cracked after magnetron sputtering process.

Sputtering target for conventional flat panel sputtering process is produced completely separated from the backing plate that supports the sputtering target in a deposition chamber. The bonding of the target blanks to a backing plate using soldering process requires low melting point solders [10, 27]. Soldering a target layer to a backing plate affects highly ITO film properties. When the bonding between the substrate and the source material is unstable during sputtering, the target cracking and eventually film contamination during deposition process can occur (Figure 1) [1, 27]. Another problem is arcing during ion beam sputtering (e.g., magnetron sputtering), which occurs as a result of an intensely focused and localized discharge sputtered by collective electron emission from a breakdown event [23, 26]. In sputtering plasma a charge can accumulate on any surface. One of the most common sources of arcs is localized charging of particles, created nodules, flakes, or impurities either on or near the surface of a target. The particle arcing events and emission during film deposition depend mostly on target density and microstructure uniformity (dopant distribution).

To eliminate previously described problems a new process of targets preparation should be used. An interesting alternative is cold spraying, one of the newest methods in thermal spraying. In cold spraying powder is deposited in solid state. Hence deposited coatings possess homogeneous structure and are free of oxidation, porosity, and new formed intermetallic phases [28–31]. Process temperature is much below melting point of sprayed material. Powder particles gain high velocity and temperature from compressed and heated supersonic gas stream in de Laval nozzle. As a working gas air, nitrogen, or helium can be used. Moreover bonding of powder particles to the substrate is provided by high kinetic energy resulting from particles supersonic velocity. In cold spraying both metal and cermet coatings (i.e., a substantially uniform mixture of two or more metallic and oxide components) can be sprayed. According to the literature [32–35] ceramic powder present in metal-ceramics mixture decreases the porosity in the coatings. This seems to be attractive in a context of ITO targets that should possess high density to maximise their life time and reduce tendency to the nodules formation.

The aim of this study was to investigate a new method of TCO target preparation using low-pressure cold spraying. Currently ITO sputtering targets are produced completely separated from the backing plates. Therefore in a second step soldering is used in order to connect the target and backing plate together. Because cold spraying does not substantially heat the powder it can be used to make targets directly on the backing plate. Moreover, cold spraying is widely industrially used to coat large areas, whereas currently used sputtering targets formed via conventional fabrication methods tend to be too small for many applications. Despite the application potential of the cold spraying method for the sputtering target production, the number of scientific articles in this field is strongly limited. To the best of our knowledge there are only two papers describing the implementation of the cold spray process in metallic sputtering target material manufacturing [36, 37]. No studies have yet been published on the manufacture of thick coating layers using cold spray deposition as transparent conductive oxides (TCOs) sputtering target materials.

As already mentioned, in magnetron sputtering process used for the TCO deposition, two types of targets are used: more expensive ceramic (In_2_O_3_-SnO_2_) or relatively cheap metallic (In-Sn alloy). In this study, for the first time, new type of target is tested, namely, target with the cermet structure in which a metal (Sn) and a metal oxide (In_2_O_3_) are mixed. Thus, the proposed sputtering target has a form of cermet Sn+In_2_O_3_ coating sprayed onto copper substrate.

## 2. Methodology and Materials

**Figure 2:**
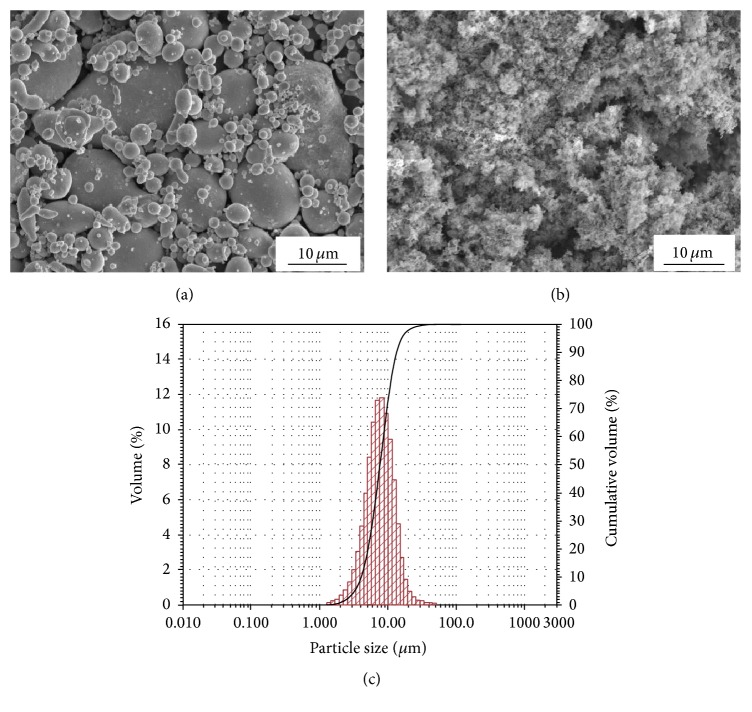
Micrographs (SEM) of tin (a) and indium oxide (b) powders used in the research and tin particles size distribution (c).

In the research two commercially available powders, spherical tin (Sn) from Libra (Trzebinia, Poland) (Figure 2(a)) and needle indium oxide (In_2_O_3_) from VWR Chemicals (Leuven, Belgium) (Figure 2(b)), were used in the spraying process. Indium oxide powder created sponge agglomerates. The granulometry measurements (Analysette 22 MicroTec plus, Fritsch, Markt Einersheim) showed that the mean particles size was 8.7 *μ*m and 0.779 *μ*m for Sn (Figure 2(c)) and In_2_O_3_ powders, respectively. A copper M1E disc with diameter of 65 mm and thickness of 5 mm was used as a substrate material.

A DYMET 413 (Obninsk Center for Powder Spraying, Obninsk, Russia) setup was used to deposit the coatings. The device includes a spraying gun with an inner gas heater and de Laval nozzle having length of 250 mm and outlet diameter of 8 mm. Air was used as the working gas to oxidize tin powder. A spraying gun was attached to manipulator holder and moved with helix path having pitch of 4 mm, so that next bead covers part of the previous one. Pure tin was in the first step used as the feedstock powder in order to optimize cold spraying process parameters. Analysing the deposition of pure tin, it was found that high preheating gas temperature causes tin oxidation. High degree of tin oxidation in final coating seems to be beneficial when we want to use it as TCO sputtering target materials. In the process of tin coating deposition, the highest deposition efficiency of wt. 73% was obtained for the following optimal parameters: (i) working gas pressure of 0.6 MPa, (ii) working gas temperature of 300°C, (iii) linear speed of 10 mm/s, (iv) powder feed rate of 50 g/min, and (v) spray distance of 20 mm. Three coating layers were successively deposited and final total coating thickness was in the range of 720–1080 *μ*m.

The cermet Sn+In_2_O_3_ coatings were sprayed using mixture of tin and indium oxide powders in volume ratio of 1 : 1. The powder mixtures were prepared using three various methods: (i) mixing in a chamber with rotation speed of 120 rpm, powder 1, (ii) milling with vibrating one-ball mill (ball diameter of 52 mm) in a chamber with lining and ball made of tungsten carbide and vibration amplitude of 10 mm, powder 2, and (iii) milling with high-energy ball mill (ball diameter of 3 mm) in a chamber with lining and ball made of zirconium oxide, ball to powder wt. ratio of 1 : 1 and rotation speed of 300 rpm, powder 3. The time of mixing and milling was 30 min.

The metallographic examinations of powders and coatings were carried out using Nikon Eclipse MA 200 optical microscope and SEM (HITACHI S-3400 N) microscope equipped with SE, BSE detectors, and EDS system for elemental analysis. The metallographic cross-section of the coatings had been etched using 1% solution of hydrogen chloride in water. For chemical characterization of samples EDX spectroscopy analysis was conducted using Phenom G2 Pro (Eindhoven, Netherlands). What is more for chosen materials X-ray diffraction analysis was performed on the feedstock powders as well as on cold spray coated specimens in the middle of its thickness. X-ray diffraction measurements were carried out using Rigaku Ultima IV Diffractometer with Cu K*α* irradiation (*λ* = 1.5406 Å) within the range from 20° to 90° in 0.02° steps with an exposure time of 4 s per point.

To examine the possibility that the cermet cold sprayed coatings may be applied as sputtering target materials, magnetron sputtering tests were conducted. Deposition process was conducted with the use of WMK-50 magnetron, which is designed to work with power densities up to 50 W/cm^2^, facilitated by a very effective cooling system. Final pressure in the NP-500 vacuum chamber was 2.66 mPa (2·10^−5^ Tr). Thin films were deposited in the atmosphere of Ar+O_2_, to oxidize tin atoms, and the gas working pressure in the vacuum chamber of 0.53 Pa (4·10^−3^ Tr). Targets were placed 75 mm from the cathode surface. The power density in the cathode was 25 W/cm^2^.

## 3. Results and Discussion

**Figure 3:**
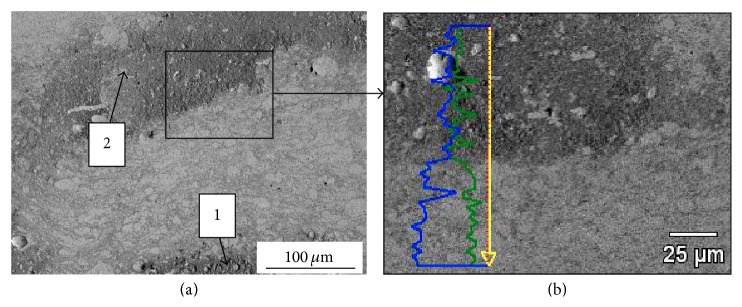
Microstructure (SEM) of tin coating (a) and chemical linear analysis (b). Tin and oxygen content are marked by blue and green lines, respectively.

Initial investigations of sprayed coatings for the purpose of comparison were carried out on those deposited with the pure tin feedstock powder. According to the literature cold spraying with tin powder is used to deposit interlayers (e.g., on polymer substrate) [38] or as a mixture with other metal for further coating modification by, for example, annealing process [39]. As it was previously demonstrated [40] low-pressure cold spraying method provides a unique possibility of producing uniformly dense pure tin and tin cermet corrosion-resisting coatings. It is well-known that tin easily oxidized when it is heated in the presence of air. Because the final goal of the research was to obtain targets for magnetron sputtering of oxide films, the process of metallic tin oxidation should be considered as an advantage. In the present study relatively high temperature of 300°C was used and thus it is possible that, despite high velocity, tin particles sprayed onto the copper substrate oxidized in the gas stream. It is also worth stressing that smaller metal particles oxidize easier [39]; therefore tin powder with relatively small size particles was used in the study. Temperature of the coating measured with thermovision camera Flir A320 during spraying was up to 111.7°C. The oxidation rate is highest next to substrate (Figure 3(a), area marked with 1) and between two deposited coating layers (Figure 3(a), area marked with 2). As the data in Figure 3(b) indicated, the proportion of tin (blue line) and oxygen (green line) between those two regions varies considerably along the line of analysis. Because all deposited coatings consist of several layers, oxide bands are visible in the coatings microstructures independently of the feedstock used.

**Figure 4:**
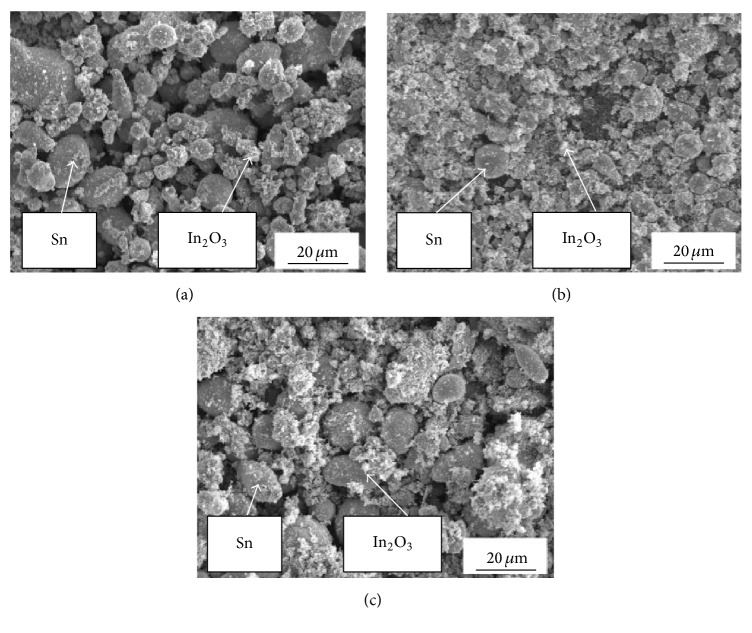
Micrographs (SEM) of feedstock prepared by mixing, powder 1 (a), milling with vibrating one-ball mill, powder 2 (b), and milling with ball mill, powder 3 (c).

**Figure 5:**
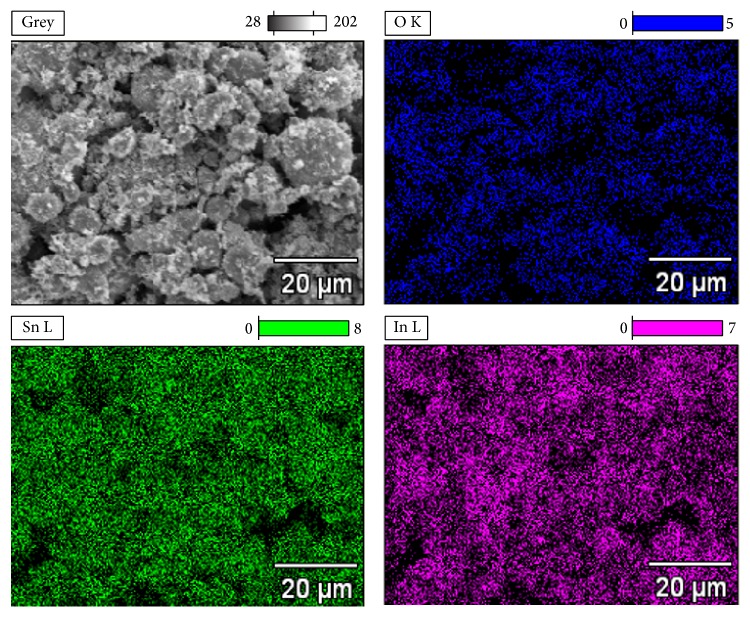
Chemical composition analysis (mapping) of powder 2.

Morphology of powders mixtures used in the research for cermet coatings production is shown in Figure 4. The main visible difference between analysed powders is distribution of indium oxide in the tin matrix. It is clear (Figure 4(a)) that powder 1 prepared in mixing chamber showed the least effective distribution of indium oxide. Ceramic particles formed agglomerates separated from the tin matrix. In the case of powder 2 (Figure 4(b)) prepared by one-ball milling, clearly improved distribution of indium oxide is observed, while powder 3 mixed in high-energy ball mill showed mixture of tin particles with intensively agglomerated tin and indium oxide (Figure 4(c)). Chemical composition analysis confirmed that powder 2 had highest distribution of indium oxide on the surface of tin particles (Figure 5), compared to other powders.

**Figure 6:**
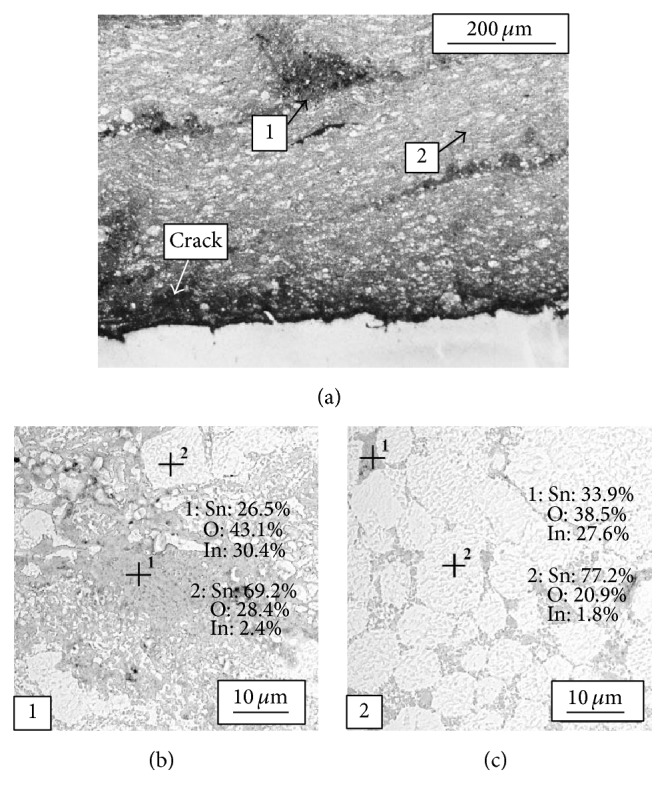
Micrographs (light microscope) of coating deposited with powder 1 (a) and chemical composition analysis (SEM-EDX) of the coating ((b), (c)).

As shown in Figure 6 coating deposited with powder 1 has high heterogeneity. The microstructure of the coating consists of highly oxidized regions with increased amount of indium and tin oxides (Figure 6(a): area marked as 1) mixed with metallic tin rich regions (Figure 6(a): area marked as 2). Chemical composition analysis confirmed higher amount of indium in highly oxidized regions up to 30.4 wt.% (Figures 6(b) and 6(c)). Regions with increased amount of Sn particles are also visible (Figures 6(b) and 6(c)). Such observed microstructural evolution is significantly affected by the process of feedstock powder preparation. As it was already described, powder 1 prepared in mixing chamber showed the least effective distribution of indium oxide in tin matrix. What is more single tin particles in highly oxidized state deposited on the substrate are clearly visible in Figure 6(a). As a result of strongly oxidized interface zone between the coating and substrate, some cracks are observed together with porosity and oblong holes.

**Figure 7:**
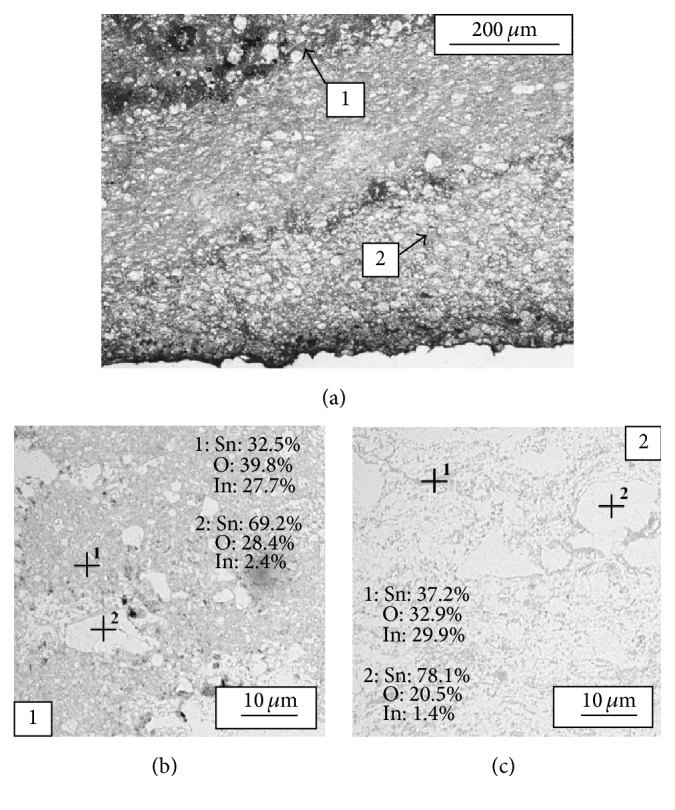
Micrographs (light microscope) of coating deposited with powder 2 (a) and chemical composition analysis (SEM-EDX) of the coating ((b), (c)).

As it was described earlier, powder 2 prepared by one-ball milling provided highest, optimal distribution of indium oxide in tin matrix (Figure 4(b)). As a result, coating with very good homogeneity of tin, tin oxide, and indium oxide was deposited. In Figure 7(a) its microstructure covering three single deposition layers is presented. It is evident that the produced coating from powder 2 was free of porosity and cracking. The coating chemical composition is presented in Figures 7(b) and 7(c). Regions with increased amount of In_2_O_3_ are locally present in the regions with increased oxidation (Figures 7(a), 7(b), and 7(c), areas marked as 1). The highest locally observed indium amount was 29.9 wt.%.

**Figure 8:**
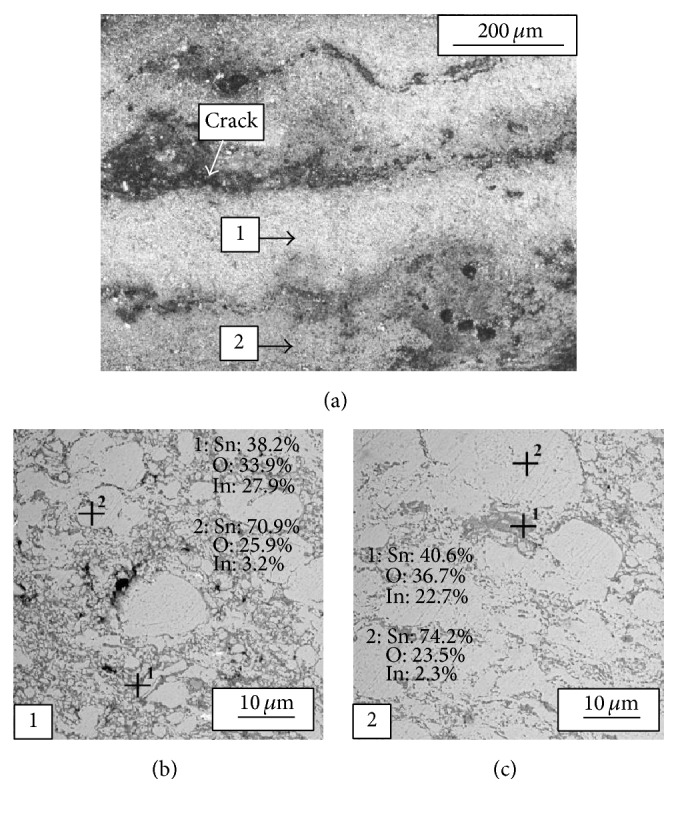
Micrographs (light microscope) of coating deposited with powder 3 (a) and chemical composition analysis (SEM-EDX) of the coating ((b), (c)).

As it was already described, powder 3 after preparation in high-energy ball mill consists of multiagglomerates of tin and indium oxide particles together with separated tin particles (Figure 4(c)). As a result, in micrographs of coating deposited with powder 3 increased amount of local regions with indium and tin oxides agglomerations is visible (Figures 8(a) and 8(b)). Furthermore, it can be seen that some tin particles crushed in high-energy ball mill were additionally intensively deformed while spraying. Porosity as well as local cracks 50–100 *μ*m thick was also observed in the coating. The coating chemical composition is presented in Figures 8(b) and 8(c).

Recently, metal-ceramic mixtures (cermets) were very often reported as cold spray source powders [28, 30–35]. The size of ceramic powder used for spraying is usually comparable to the size of metal powder and for this reason they do not form separate agglomerates. Thus, obtained in such a way coatings have uniform distribution of ceramic particles [35, 40].

Summarizing the above observations, the least satisfactory coating structure was obtained with powder 3, as a result of intensive crushing of the tin powder by milling balls. The most homogeneous and satisfactory structure showed coating deposited with powder 2, as it was free of pores and cracks and with the most regular distribution of ingredients: tin, tin oxide, and indium oxide. Since the high degree of tin oxidation in final coating seems to be beneficial when we want to use it as TCO sputtering target materials, powder 2 was subjected to further modification in the furnace. To this end, powder 2 was additionally heat treated in the temperature of 220°C for 6 hours in the atmosphere of air to oxidize tin particles and provide higher amount of tin oxide in the coating. The obtained, in this way, powder (HT powder 2) was used for coating deposition.

**Figure 9:**
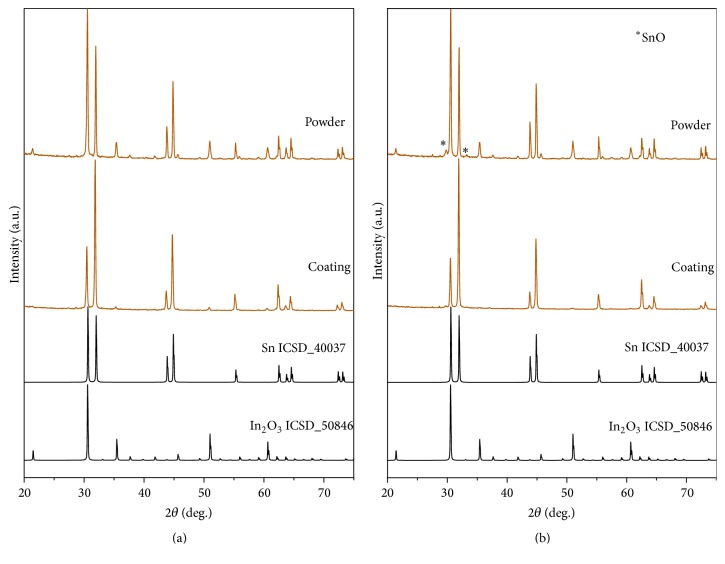
XRD diffraction patterns of coatings and initial powder feedstocks: (a) powder 2 and coating and (b) HT powder 2 and coating.

In order to identify the phase composition of powders considered as the most suitable for TCO sputtering target production, XRD measurements were performed (Figure 9). The analysis of the diffraction patterns of powder 2 and HT powder 2 indicated typical reflections for tin with a tetragonal structure (*β*-Sn) together with similarly intensive reflections for cubic indium oxide. For HT powder 2 additionally low intense diffraction peaks corresponding to SnO were indicated, confirming high degree of tin oxidation after heat treatment. In both final coatings (produced with powder 2 and HT powder 2), XRD measurements indicated significant decrease of indium oxide in relation to tin. No additional diffraction peaks reflecting any tin oxide were seen on XRD diffractograms of coatings.

**Figure 10:**
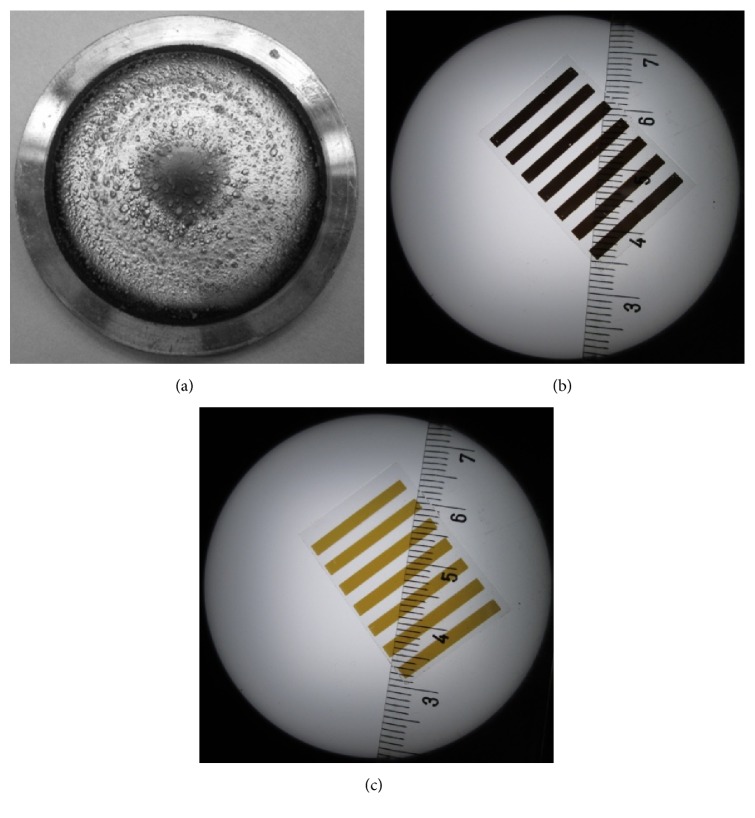
Results of magnetron sputtering process: free from damage target after the preliminary sputtering test (a), nontransparent TCO film coating (b), and transparent TCO film coating (c).

To examine the possibility that the cermet cold sprayed coatings prepared with powder 2 and HT powder 2 may be applied as sputtering target materials, magnetron sputtering tests were conducted. After preliminary tests, it is encouraging that an actual thin film is able to be formed. Targets produced with cold spraying method worked very well (Figure 10(a)), compared to commercially available targets (Figure 1). After the process there were no signs of damage on the target surface and visible surface roughness resulted from cold spraying process (Figure 10(a)). Hence, targets showed yield improvement and longer serviceable life. However, thin films produced from target prepared with powder 2 were nontransparent and nonconducting. On the other hand, target produced from HT powder 2 showed some satisfactory results. Initially produced film by using HT powder 2 had the thickness of 200 nm and was fully transparent (Figure 10(b)). However, its resistance 1 MΩ (sheet resistance of 1·10^5^ Ω/sq) was unsatisfactory. Further tests, using longer deposition time, are enable of obtaining thicker film with satisfactorily lower resistance 200 kΩ (sheet resistance of 2·10^4^ Ω/sq), although much less transparent (Figure 10(c)). Clearly, further optimization of the deposition conditions is required. However, very encouraging results up till now advise for future fabrication of TCO films using targets produced by cold spraying.

## 4. Conclusions

In the article new cermet Sn-In_2_O_3_ targets produced with cold spraying for magnetron sputtering process of TCO thin films are presented. The analysis of their microstructures showed that tin particles oxidize easily while spraying with air stream. However, single tin particles remain in the target and thus have to be oxidized during magnetron sputtering. Microstructure of the coatings depends strongly on the feedstock powder mixture preparation method. It was found that powder produced by milling in one-ball mill enables producing of coating with good homogeneity of metallic tin, tin oxide, and indium oxide. In order to increase degree of tin oxidation favourable for TCO sputtering target materials production, this powder was subjected to further modification in the furnace and then used as a feedstock for spraying. To examine the possibility that the chosen cold sprayed coatings may be applied as sputtering target materials, first preliminary magnetron sputtering tests were conducted. Very encouraging results up till now advise for future fabrication of TCO films using targets produced by cold spraying. However, further development and testing are required.
