# Corrigendum to “Microscopic Examination of Cold Spray Cermet Sn+In_2_O_3_ Coatings for Sputtering Target Materials”

**DOI:** 10.1155/2017/3107374

**Published:** 2017-10-25

**Authors:** M. Winnicki, A. Baszczuk, M. Rutkowska-Gorczyca, M. Jasiorski, A. Małachowska, W. Posadowski, Z. Znamirowski, A. Ambroziak

**Affiliations:** Wrocław University of Technology, Wyb. Wyspiańskiego 27, 50371 Wrocław, Poland

In the article titled “Microscopic Examination of Cold Spray Cermet Sn+In_2_O_3_ Coatings for Sputtering Target Materials” [1], an acknowledgment should be added as follows.

This paper is based on the research supported by the Foundation for Polish Science (FNP).
